# Lithium first choice: not merely first line

**DOI:** 10.1192/j.eurpsy.2026.10396

**Published:** 2026-07-31

**Authors:** M. Bauer

**Affiliations:** Psychiatry and Psychotherapy, University Hospital Dresden, Technische Universität Dresden, Dresden, Germany

## Abstract

**Abstract:**

More than 75 years after the breakthrough in identifying lithium salts as having antimanic and prophylactic activity, modern research continues to confirm lithium’s position at the forefront of the treatment of bipolar disorder. Undoubtedly, lithium is an essential medication for patients with mood disorders and represents the most valuable treatment option in the long-term management of bipolar disorder. When used correctly, lithium unquestionably produces the most dramatic benefits of any medication in psychopharmacology. Since its introduction to modern psychiatry in 1949, basic and clinical research has uncovered many new aspects of its use in psychiatry, including antisuicidal and antidepressant properties. Lithium is considered the first-line “gold standard” medication for the maintenance treatment of bipolar disorder with serum lithium levels ideally in the range of 0.6-0.8 mmol/L. Over the past three decades, there has been increasing recognition of lithium’s efficacy in conferring long-lasting mood stability, a perception of its capabilities that is reflected in clinical practice guidelines worldwide. Recent independently published meta-analyses included all available placebo randomized controlled trials (RCTs) and reached similar conclusions: lithium was statistically significantly superior to placebo for the prevention of affective episodes of any polarity, manic episodes, and, depending on the methodology used, depressive episodes. Although RCTs are fundamental for demonstrating the efficacy and safety of medications, their results do not reflect the entire population of patients with bipolar disorder seen in clinical practice. Given the limitations and uncertainties of RCTs, it is important to compare evidence from RCTs with evidence from observational studies of patients treated in so-called real-world clinical settings, e.g. using a naturalistic design and typically including a wide range of patients. A systematic review of nonrandomized controlled observational studies by a task force of the International Society for Bipolar Disorders (ISBD) found that in eight of nine studies identified, with a total of more than 14 000 patients, maintenance lithium therapy was associated with improved outcomes compared with another mood stabilizer in monotherapy.

**Disclosure of Interest:**

M. Bauer Consultant of: In the past 3 years, MB has served as an advisor to Alfred E. Tiefenbacher GmbH Co. KG, COMPASS Pathfinder Ltd., GH Research, MedEd-Link Inc., Janssen Global Services, LLC, Livanova, Mindforce Game Lab AB, and Lilly.

